# Perceptions of Community Involvement in the Peruvian Mental Health Reform Process Among Clinicians and Policy-Makers: A Qualitative Study

**DOI:** 10.15171/ijhpm.2019.68

**Published:** 2019-08-21

**Authors:** Jose A. Arriola-Vigo, Jeffrey G. Stovall, Troy D. Moon, Carolyn M. Audet, Francisco Diez-Canseco

**Affiliations:** ^1^Department of Psychiatry and Behavioral Sciences, Vanderbilt University Medical Center, Nashville, TN, USA.; ^2^Department of Pediatrics, Division of Infectious Diseases, and Vanderbilt Institute for Global Health, Vanderbilt University Medical Center, Nashville, TN, USA.; ^3^Department of Health Policy, and Vanderbilt Institute for Global Health, Vanderbilt University Medical Center, Nashville, TN, USA.; ^4^CRONICAS Center of Excellence in Chronic Diseases, Universidad Peruana Cayetano Heredia, Lima, Peru.

**Keywords:** Mental Health Reform, Community Engagement, Community Mental Health, Health Policy, Peru

## Abstract

**Background:** The global burden of mental health conditions has led to the implementation of new models of care for persons with mental illness. Recent mental health reforms in Peru include the implementation of a community mental health model (CMHM) that, among its core objectives, aims to provide care in the community through specialized facilities, the community mental health centers (CMHCs). Community involvement is a key component of this model. This study aims to describe perceptions of community engagement activities in the current model of care in three CMHCs and identify barriers and potential solutions to implementation.

**Methods:** A qualitative research study using in-depth semi-structured interviews with clinicians from three CMHCs and with policy-makers involved in the implementation of the mental health reforms was conducted in two regions of Peru. The interviews, conducted in Spanish, were digitally recorded with consent, transcribed and analyzed using principles of grounded theory applying a framework approach. Community engagement activities are described at different stages of patient care.

**Results:** Twenty-five full-time employees (17 women, 8 men) were interviewed, of which 21 were clinicians (diverse health professions) from CMHCs, and 4 were policy-makers. Interviews elucidated community engagement activities currently being utilized including: (1) employing community mental health workers (CMHWs); (2) home visits; (3) psychosocial clubs; (4) mental health workshops and campaigns; and (5) peer support groups. Inadequate infrastructure and financial resources, lack of knowledge about the CMHM, poorly defined catchment areas, stigma, and inadequate productivity approach were identified as barriers to program implementation. Solutions suggested by participants included increasing knowledge and awareness about mental health and the new model, implementation of peer-training, and improving productivity evaluation and research initiatives.

**Conclusion:** Community engagement activities are being conducted in Peru as part of a new model of care. However, their structure, frequency, and content are perceived by clinicians and policy-makers as highly variable due to a lack of consistent training and resources across CMHCs. Barriers to implementation should be quickly addressed and potential solutions executed, so that scale-up best optimizes the utilization of resources in the implementation process.

## Background

Mental health, neurologic, and substance use disorders account for nine of the top twenty leading causes of years lived with disability and 10% of the global burden of disease.^1-3^ Mental health disorders are disproportionally represented among the poor, either as a result of a drift by those with mental health problems toward more socially disadvantaged circumstances or because of less access to healthcare compared to the general population.^3,4^ Patients with mental health disorders are six times more likely to use emergency departments compared with patients without psychiatric conditions.^5-8^

In recent years, several countries have implemented models of care to promote, prevent, and treat patients with mental health conditions in the community, moving away from the tertiary healthcare facility-based model.^9-12^ In this context, the World Health Organization is actively supporting the community mental health model (CMHM) as an effective approach to provide mental healthcare.^13,14^ Community mental healthcare is defined as comprising the principles and practices needed to promote health for a local population by addressing their needs in an accessible and acceptable way; building on the goals and strengths of people with mental conditions; promoting a wide network of supports, services and resources of adequate capacity; and emphasizing services that are both evidence‐based and recovery‐oriented. ^15,16^

Countries in Latin America have begun to implement the CMHM.^17,18^ CMHM implementation in Peru started in 2012 as part of mental health reform intended to address the high prevalence of mental illness in Peruvian society, where one out of 5 persons suffers from a mental disorder.^19^ This pattern is further exaggerated in the Peruvian highlands and jungle areas, where the life time prevalence of any psychiatric condition is 30% and 40%, respectively.^20,21^

The Peruvian mental health reform has entailed a significant shift in the approach and priorities related to mental healthcare. Encouragement to use tertiary mental health institutions declined, and mental healthcare transitioned into the CMHM.^22^ The CMHM aims to build a healthcare network with particular emphasis on providing mental healthcare in the community through specialized outpatient facilities, known as community mental health centers (CMHCs). CMHCs are predominantly staffed by psychiatrists, primary care physicians, psychologists, nurses, therapists, and social workers; and an aim to provide psychotherapy, rehabilitation services, and medication management services for patients with mild to moderate substance use and mood disorders, schizophrenia, and developmental disorders throughout the life span.^22,23^

**Figure 1:**
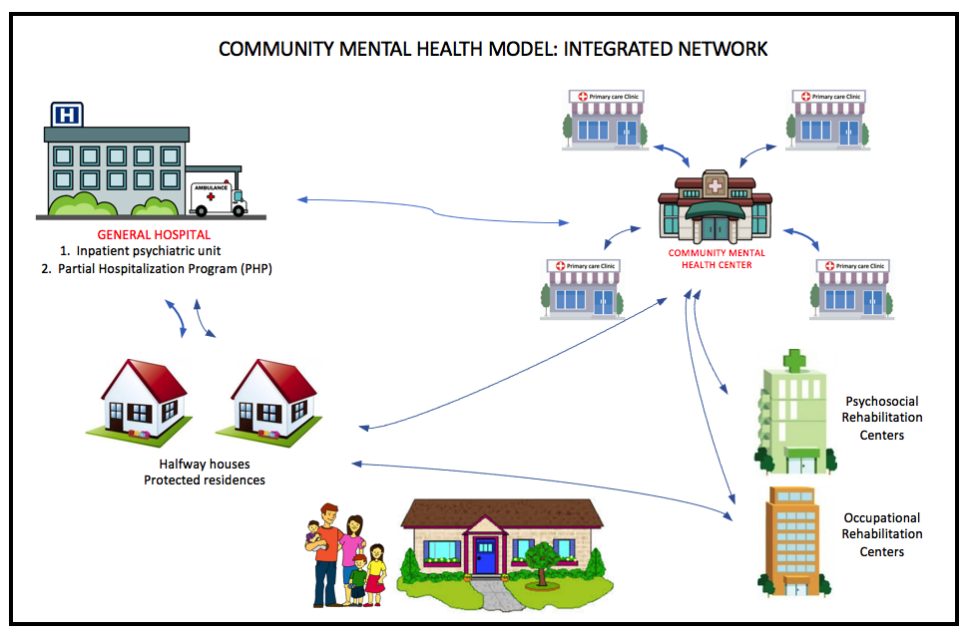
Integrated Mental Health System: CMHM. Abbreviation: CMHM, community mental health model.^25^

In addition to CMHCs, four mid level mental healthcare facilities have been proposed to complement a community-based mental healthcare network: (*a* ) protected homes or halfway houses for patients discharged from psychiatric hospitals lacking family support; (*b* ) protected residences for patients who suffer from disabling sequelae; (*c* ) specialized psychosocial rehabilitation centers to help patients recover their autonomy and provide support to families as patients reintegrate into society; and (*d* ) occupational rehabilitation centers which are designed to help patients recover or improve their job skills^22,24,25^ (Figure 1).

Consistent financial resources and political will have been critical for the ongoing mental health reform. Although legislation changes have occurred since 2004, a crucial step was taken in 2014 with the creation of a pay-for-performance budget for Control and Prevention in Mental Health (PpR 131) by the Peruvian Ministry of Economy and Finance. This budget has translated into a 10-year commitment for allocation of resources to mental health activities based on the fulfillment of measurable indicators.^22^

As of December 2017, a total of 31 CMHCs have been built in Peru, with an ultimate goal to reach 281 CMHCs by 2021.^24-27^ CMHCs play a pivotal role in this mental health network, through coordination of care with specialized and non-specialized institutions, and community organizations. Community involvement stands out as a main priority for the CMHCs. Community involvement entails to have people in the community engaged in the care of the mentally ill and aims to be a key component throughout the continuum of care. The CMHM has proposed to strongly emphasize extramural activities in the CMHCs, which may include home visits, workshops, campaigns and active collaboration with community leaders.^28^

In the declaration of Alma-Ata in 1978, community participation was supported as a fundamental component of primary healthcare. Since then, the term community engagement, as opposed to participation, emerged from the field of health research to describe a deliberate integration of communities into the design, implementation and monitoring in healthcare.^29-31^ Although the terms community engagement, participation and involvement in health development may have slight differences, they are currently used interchangeable to describe an imaginative new approach which seeks to bring together the formal, professional health structure and local people with their knowledge and resources.^32^

Community engagement activities have been shown to be cost effective, provide a better understanding of a community’s health status, and increase overall knowledge about health. However, the definition of specific activities being implemented is not standardized due to variability in a given community’s resources and/or the barriers to implementation for each specific environment.^32^

The Peruvian guidelines for the CMHM define community engagement and mobilization as processes of committed and responsible participation of the members and leaders of the community and its organizations. Although the overarching goals of community engagement are described in the national guidelines, the activities proposed to be performed in the field are limited to home visits, workshops to promote mental health awareness, and peer-support groups, without definitions or standardized parameters for their implementation and roll-out.^24,25^ Furthermore, quality control and evaluation programs intended to optimize the utilization of resources in the Peruvian mental health reform are limited.^28^

We describe perceptions of community involvement activities based on the continuum of care in the CMHCs, thus provide a preliminary assessment of program implementation of the CMHM in Peru. Our study aims to characterize perceptions of community involvement activities in the CMHCs as part of the current mental health reform, and to identify barriers to implementation and potential solutions, with the ultimate goal to define best practices in the implementation process.

## Methods

### Design

A qualitative research study using in-depth semi-structured interviews was conducted. The study adhered to principles of grounded theory applying a framework approach.^33^

### Participants and Setting

**Figure 2:**
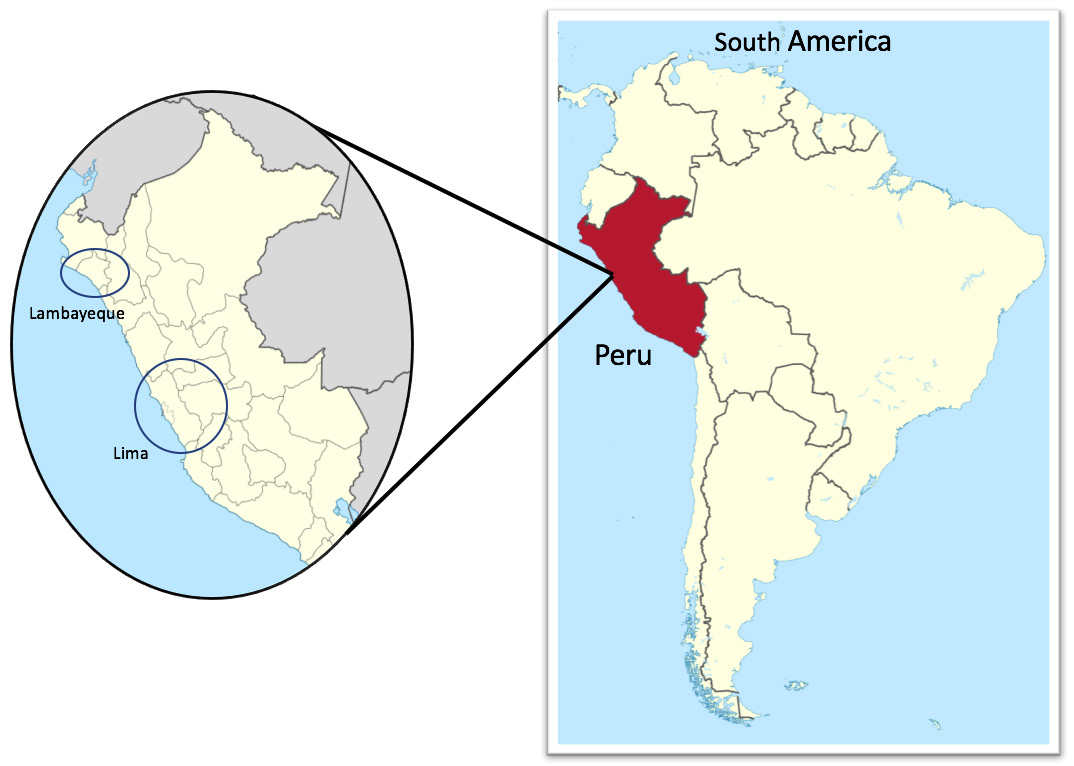
Location of CMHCs Included in the Study. Abbreviation: CMHCs, community mental health centers.

Participants were recruited through an informational talk presented at three CMHCs, two in Lima, the capital of Peru, and one in Lambayeque region, northern Peru (Figure 2).

The CMHCs have between 14 and 20 employees each. Staff members are clinicians, including psychiatrists, primary care physicians, psychologists, nurses, social workers, occupational and language therapists; and support personnel including technicians, administrative and security staff. Only clinicians were included in the study because they are expected to lead and organize the community engagement activities.^28^ After the informational talk, clinicians interested in participating in the study provided their names and were later approached separately to schedule an interview.

Four policy-makers involved in the implementation of mental health reforms were also included in the study. Policy-makers represent the major health institutions that have collaboratively worked to carry out the implementation of mental health reform. These institutions included a regional branch of the Ministry of Health (MoH) in the northern Peru (1 participant), a National Institute of Mental Health (1 participant), and a collaborating non-profit organization (2 participants).

While participation of service users is crucial in service implementation research, the authors decided not to include service users at this stage as most CMHCs had been recently implemented. As such, knowledge about community engagement activities among them would have been scarce at the time. Participation of service users will be critical for subsequent follow-up studies.

### In-Depth Semi-structured Interview Procedures

One-on-one in-depth semi-structured interviews were held in a private location with each participant. The theory of data saturation was used to define our final sample size, this theory stipulates that it is necessary to interview people until no new information relevant to the goal of the study is revealed during the structured interviews.^33^

Interviews lasted between 35 and 70 minutes. The interviews began with an introduction describing the purpose, recording process, and confidentiality of the study. The interviews were conducted in Spanish, the native language of participants. The interviews were recorded using a digital audio recorder, with the voice files subsequently transcribed by an independent transcriber. Subsequently, identifying information, including mention of staff member or names were removed. The transcriptions were then reviewed to assure quality, confidentiality, and reliability, as well as for formatting for coding. This information was then saved in a secure server at Vanderbilt University Medical Center, Nashville, TN, USA.

The interviewer followed an interview guide developed before the study initiation. Two interview guides were developed, one for clinicians and one for policy-makers to facilitate data collection. The interview guides were pilot-tested with a Spanish-speaking Peruvian psychiatrist to assure quality and clarity of the questions. The interview guides were created directly in Spanish. Questions were divided in three sections that included demographics, inter-professional collaboration, and community engagement activities. Each interview guide had a total of 19 questions, of which the community engagement section included four primary questions (Box 1). Each question included more specific follow-up prompts that the interviewer could use to obtain more detailed information.

**Box 1. Questions Related to Community Engagement Activities in the Interview Guide**

1. What activities does this CMHC do to promote community involvement?
2. Can you explain how each of these activities is performed in the CMHC to promote community involvement? Are these effective? If not, please proceed to question 3, otherwise proceed to question 4.
3. What prevents the CMHC from effectively engaging with the local communities?
4. Would you propose changes in the way that this CMHC engages with the local communities? If so, what changes would you propose?

Abbreviation: CMHC, community mental health center.

### Qualitative Analysis

Each sentence was treated as a separate quote and coded using a hierarchical coding system. Before the developing of codes, the interviews were formatted to be imported to MAXQDA 2018 version software used for data analysis. The coded data were used to summarize the quotes and identify themes using principles of grounded theory applying a framework approach as described by Glaser and Strauss.^33,34^ Grounded theory aims to generate theories from the collected data, which are summarize into different themes. Initial open coding generated significant amount of codes which were refined as the data analysis progressed. The coding system for community engagement activities included inductive codes for major categories based on the description of each activity, and hypothesized connections between them. Each category was subdivided and expanded further based on the information obtained in the study. The coding process was conducted in Spanish, and then translated into English. Delaying the translation into English was purposefully done to avoid losing themes, signals or meanings associated with quotes. The total number of codes (3 codes, and 23 sub-codes) were used 1301 times to describe the themes present in 3540 quotes (Supplementary file 1).

## Results

### Participant Characteristics

A total of 25 participants were included (17 women, 8 men), who were full-time employees (21 healthcare professionals, 4 policy-makers). Table 1 provides an overview of the participants’ occupation. All participants had higher education including bachelor’s degree only (10), bachelor’s and master’s degree (9), or Medical Doctor (MD) degree (6).

**Table 1:** Final Study Sample - Participants’ Occupation

**Occupation**	**N**
Nurses	5
Psychiatrists	6
Occupational and language therapists	5
Psychologists	7
Social Workers	2
Total	25

The policy-makers that participated in the study included a director of community health in a National Institute of Mental Health, a director of the regional mental health program in the MoH, a director of project development and cooperation, and a consultant psychiatrist in a non-profit organization. The former two were involved in the development of the pilot project, and then implementation of the new model of care in a regional and national level, respectively. The latter two were part of a non-profit organization which collaborated with the Peruvian MoH throughout the implementation of the CMHM in a national level.

The results were divided into 3 categories within the community engagement umbrella: (1) current activities performed; (2) barriers; and (3) proposed solutions.

### Community Engagement Activities

**Figure 3:**
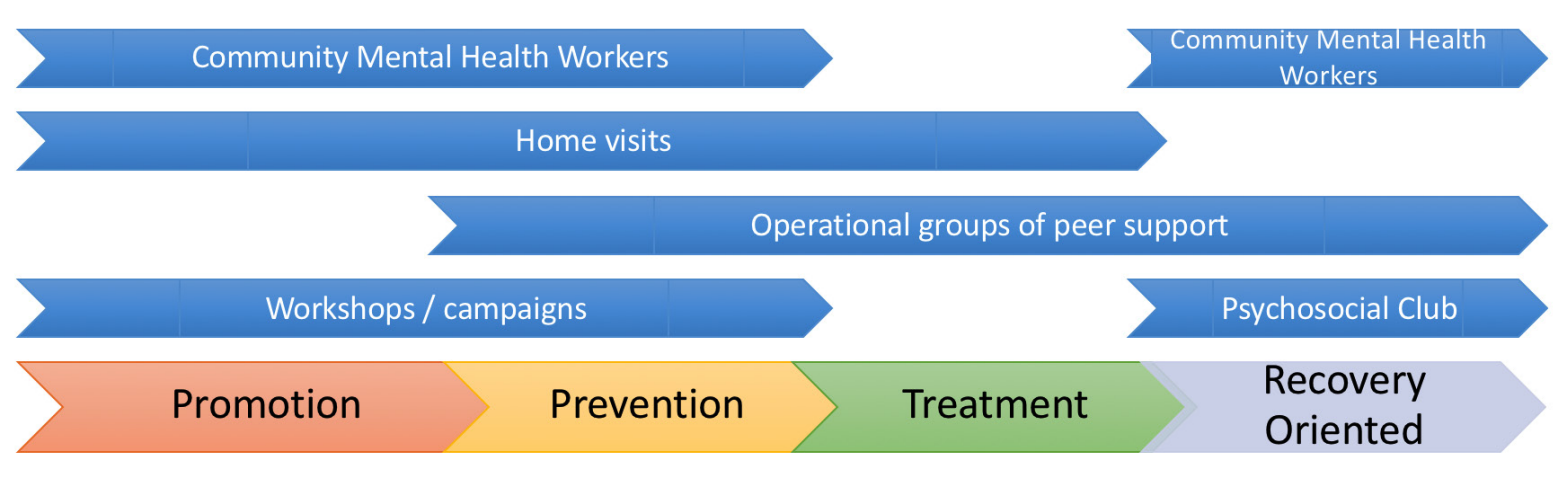
Community Engagement Activities in the Continuum of Care.

The description and definition of 5 activities emerged from the interviews: (1) Employing community mental health workers (CMHWs); (2) home visits; (3) mental health workshops and campaigns; (4) psychosocial clubs; and (5) operational groups of peer support. Each of these activities aims to target different stages in the continuum of care.^35^ A pictorial representation of these activities in the context of the continuum of care was drawn from the interviews and is shown in Figure 3.

### Community Mental Health Workers

Employing CMHWs was found to be one of the most consistent interventions in the CMHCs. Participants reported that CMHWs are currently in different stages of training, and that most CMHCs have already started the recruitment process. Clinicians indicated that CMHWs are community leaders who do not necessarily hold a health-related degree. CMHWs receive training about promotion, prevention, and recovery-oriented activities for people with mental health conditions. A nurse described the role of CMHWs by saying:

*“They help us mainly with promotion and prevention activities, they know the catchment area, and are able to follow up with patients. For instance, if we have a patient in the area, they already know him, and take us quickly to do a home visit…”* (N1/CMHC1).

The training structure and curricula for CMHWs vary in each CMCH according to what clinicians consider a priority for their target community. Two training programs for CMHWs were described: (1) An intensive weekly training program followed by less frequent booster sessions, (2) Only a weekly training program for a set number of weeks. Participants did not specify length of each training session.

CMHWs are recruited through meetings with the community. Their work is voluntary and uncompensated, which appears to impact their motivation to remain engaged over time. This was described by a participant social worker:

*“…Since last year, we have recruited CMHWs from the community to get voluntary support, however, slowly we have seen that they have other activities to attend. They do not feel motivated because they expect to receive something in exchange, but this is a voluntary job where they are not compensated”* (T1/CMHC1).

The recruitment and training process for CMHWs tends to move faster when communities are proactive and organized, which also evidences the strong motivation of their members to do good for their communities. This was evidenced by one policy-makers who said:

*“Community organizations in …(District) are impressive, they have leaders with a historical tradition, they have made significant progress. I have to tell you, CMHWs and community leaders are working with family associations, clients…”* (PM4).

### Home Visits

Participants reported that home visits were performed in all CMHC represented in the study. The majority of the home visits were conducted predominantly by nurses and social workers; in difficult cases a psychiatrist would participate. The goal of the home visit includes promotion, prevention and treatment interventions, particularly when problems with treatment compliance and appointment attendance arise. Home visits are identified as a fundamental activity in the new model of care described by one of the policy-makers:

*“It is not only giving an appointment, it is that if you don’t show up, it is not that I have free time. It is that I am concerned that you have not showed up and I manage to try to find you at home…”* (PM3).

Participants also identified home visits as an activity for exploring how patients and their families live, and how staff members can help to improve the home environment to promote good mental health.

### Mental Health Workshops and Campaigns

Mental health workshops and campaigns were widely seen as productive and necessary activities to educate patients and their community on promotion and prevention of mental health conditions. Workshops were defined as group activities with patients and their families, where they discuss relevant mental health topics. One participant commented:

*“We have an anxiety workshops, and we invite clients’ families. Patients and their families come and learn more about the disease and condition that their family member is struggling with”* (N2/CMHC1).

Participants described mental health campaigns as collaborative psycho-education activities between the CMHC and community-based organizations, schools, primary care centers, and municipalities; with particular emphasis on mental health promotion and prevention as well as publicity for the CMHC. A language therapist said:

*“We invite schools, and we emphasize promotion of mental health and performed a screening of all students, …, because sometimes they may have a learning disability or language problems that it is not easy to recognize. Using this approach, we are able to screen kids and adolescents with language or learning problems, and then make the appropriate referral if needed”* (T1/CMHC3).

Nurses, social workers, and psychologists commonly lead and organize the campaigns and workshops. However, the participation rates of psychiatrists and language and occupational therapists were low.

### Psychosocial Clubs

Psychosocial clubs were described as social activities for people with psychotic disorders, mainly schizophrenia, who have developed significant insight on their condition and symptoms. Clinicians reported that psychosocial clubs entailed activities such as going to the beach or the movie theater, participating in playing sports, or simply socializing with peers. These aim to assess patients’ coping skills in social environments allowing healthcare professionals to provide supportive psychotherapy, or interventions in real life activities. Nurses and social workers in conjunction with patients’ families lead psychosocial clubs and decide the nature and schedule of activities. One participant nurse shared an example of the value of the psychosocial clubs for patients:

*“There was a client who attended a university meeting, and she presented and shared her experience about the implementation of the first psychosocial club in this center. When she started attending the psychosocial club, she was very introverted and shy, however she was then excited about participating in this meeting and presented her experience, she did very well!”* (N1/CMHC1).

As depicted in Figure 3, psychosocial clubs are recovery-oriented activities that aim to reintegrate patients into society.

### Operational Groups of Peer Support

A few participants reported that peer support groups were inconsistently implemented and they aim to provide patients with a safe environment to share their experiences about mental illness, normalize their symptoms, and learn coping skills from their peers. This model is an example of a recovery-oriented intervention that is not only directed for people with substance use disorders, but also people with mood or psychotic disorders. One participant nurse mentioned:

*“It (peer support groups) allows them to share their experiences with others, they talk about their diagnoses and problems. Some of them are in the recovery phase, whereas others may have just been diagnosed with a mental illness”* (N1/CMHC2).

As shown in Table 2, community engagement activities are conducted inconsistently across the CMHCs included in the study.

**Table 2:** Community Engagement Activities Performed in Each CMHC

	**CMHC #1**	**CMHC #2**	**CMHC #3**
CMHWs	+	+	+/-
Home visits	+	+/-	+/-
Workshops/campaigns	+	+/-	+/-
Psychosocial clubs	+	+	+/-
Groups of peer support	+	-	-

Abbreviations: CMHWs, community mental health workers; CMHC, community mental health center. (+) = performed regularly; (+/-) = performed intermittently, less frequently than expected; (-) = not performed at all.

### Barriers to Model Implementation

Participants reported that implementation of community engagement activities was primarily limited by: (1) inadequate infrastructure and financial resources, (2) lack of knowledge about the model and CMHCs, (3) unclear catchment area, (4) stigma, and (5) inadequate productivity approach.

### Inadequate Infrastructure and Financial Resources

Healthcare workers identified lack of space in the CMHC as a limiting factor to expand their community engagement outreach activities. More patients and their families have become interested in participating in workshops and psychosocial clubs, resulting in difficulties accommodating them in the CMHCs. Participants reported that financial and logistical resources to conduct home visits or psychosocial club activities are limited. Financial burden related to extramural activities and a lack of transportation were described as barriers to implementing community engagement activities. Several participants pointed out that healthcare workers spend their own money to conduct these activities. In this context, some centers decided to limit and decrease the number of extramural activities. One psychiatrist commented:

*“We could do more activities, we could do extramural activities more often, but this is when the financial issues come again, everything we do comes out of our pockets, it is as simple as that”* (MD1/CMHC1).

### Lack of Knowledge of the New Model of Care

Most participants reported that they had a limited amount of knowledge about the CMHM before starting to work in the CMHC. There are still healthcare professionals in the CMHC and in the primary care clinics that were not at all familiar with the new model of care. The lack of knowledge of the CMHM results on clinicians of CMHCs and primary healthcare clinics not being aware of interventions to be emphasized in the new model of care, as such, time, resources, and services are not invested efficiently on community involvement activities. One occupational therapist said:

*“Even though the plan to engage the community has changed over time, there has not been a clear approach of how community activities should be conducted. Most professionals had a vague idea about the community mental health model, and despite the support from (NAME) Specialized Mental Health Institution, we were not certain where we were going to”* (T2/CMHC1).

### Poorly Defined Catchment Area

Participants reported that the catchment area for each CMHC is unclear due to the scarcity of the centers. Healthcare professionals recognized that having an unclear catchment area has resulted in having an overwhelming number of patients and inability to apply the CMHM appropriately because of the location of patients’ homes in relation to the CMHC. Having a poorly defined catchment area has led CMHCs to provide services to people outside the district or city where the CMHC is located, resulting in inefficient utilization of resources. This barrier was identified by all CMHCs included in the study. One of the participant nurses said:

*“This district only has three hundred and fifty thousand people, therefore there should be at least three centers here, however there is only one. We are trying to provide care for them, but distance is the main barrier. We can’t follow them up as they live too further away and we do not have our own transportation”* (N1/CMHC1).

### Stigma

Stigma associated with mental health was present not only in the community, but also among healthcare professionals in the primary care clinics. Participants explained that patients were reluctant to seek mental healthcare or participate in community engagement activities coordinated by the CMHCs due to concerns about being treated differently if their friends find out that they have a mental illness. In fact, the community was initially opposed to having a CMHC in the area.

Primary care providers commonly refer patients with mental health conditions prematurely because of misconceptions of them being dangerous or having poor prognosis secondary to their mental illness. Premature referrals result on overloading CMHCs and tertiary mental health facilities with patients who could be treated in a primary care clinic.

A policy-maker noted resistance from healthcare professionals of the referring hospital and primary care center:

*“We have to fight the resistance from the hospitals to articulate this network. There are all kinds of barriers in the general hospitals, starting from philosophical and ideological conflicts from hospital directors and psychiatry and psychology division chiefs, they have old models and they are afraid of the new community model…”* (PM4).

### Inadequate Productivity Approach

Productivity was defined by participants as the number of patients expected to be seen based on the number of hours devoted to clinical activities. It was reported that the current productivity approach is based on number of patients projected to be seen in intramural clinical activities, and do not account for additional time spent in transportation, phone calls and coordination of care associated with most extramural community engagement activities. Participants reported that the frequency of community engagement activities has decreased over time as a result of the current inadequate productivity evaluation, which emphasizes intramural and office-based activities. Community engagement activities are perceived by supervising clinicians as not an efficient use of their time, which results in perceived low productivity and subsequently in the potential for payment cuts. One clinician said:

*“I just do a few extramural activities, and I can’t even schedule them consistently on a weekly basis because if I do them weekly, I somewhat lose productivity hours in the center, and this is a problem, …, therefore this does not support our productivity goals”* (T2/CMHC1).

Proposed Solutions to Implementation

Despite the growing awareness of barriers, participants also proposed potential solutions to facilitate community engagement activities in the new model of mental healthcare. Five major themes emerged including, (1) increasing knowledge about the CMHM among general practitioners, (2) implementing peer-training on community engagement activities, (3) increasing awareness for mental health in the community, (4) improving the productivity evaluation and organizational structure of the CMHCs, and (5) promoting research to assess impact of the new model of care.

### Increase Knowledge About the CMHM

Increasing awareness about the CMHM among healthcare professionals in the primary care clinics, general hospitals and community-based organizations was identified as a potential solution to fight stigma and to increase community involvement in the new model. One participant nurse reported:

*“I would propose to promote awareness in the CMHC. Maybe, providing more training to general practitioners, and also having the Ministry of Health getting more involved in creating awareness of the new model of care. The Ministry of Health should explain this model to them and inform how the care should be delivered…”* (N2/CMHC3).

Participants also proposed incorporating community health training into university curricula for healthcare providers, which would facilitate the introduction of the new model. These interventions would result on reducing stigma and resistance. A policy-maker said:

*“First, we should change the curricula of the psychiatry, psychology, and medicine training. It should not happen that psychiatry residents spend three years in (NAME) Mental Health Institute, and only 3 months, as it is now, in the Community Mental Health Centers”* (PM4).

### Implement Peer-Training on Community Engagement Activities

Participants also proposed implementing continuous and consistent peer-training strategies to allow established CMHCs model and teach how to conduct community engagement activities to newly formed centers. Participants identified this strategy as a valuable activity to standardize mental health services nationally. Peer-training and demonstrative activities were conducted during initial stages of the implementation process; however, these activities are infrequently happening in most centers. A participant nurse said:

*“We need training activities more consistently, maybe somebody from the capital. We have two years already, and we have not had staff from Lima come to train and coach us. They should tell us ‘look guys, this is what it is going well and this is what is not going well.’ We feel a little bit isolated and too far away…”* (N1/CMHC2).

Increase Awareness for Mental Health in the Community

Increasing awareness for mental health was identified as a potential solution to fighting stigma and to promote participation in community engagement activities. Participants proposed providing more resources for continuous publicity and mental health campaigns with the ultimate goal to overcome resistance to mental healthcare. For instance, a participant nurse said:

*“We should slowly start to involve them (community), and we will then have more patients who live close to the CMHC. Recently, we have heard that they are happy to have a CMHC here”* (N1/CMHC1).

A social worker recommended:

*“We should work more on psychoeducation with the family, we see that sometimes the family is moving away instead of moving closer (to the CMHC)”* (T1/CMHC1).

Improve Productivity Evaluation and Organizational Structure in the CMHCs

The productivity approach was proposed to emphasize extramural activities rather than office-based care. Participants mentioned that healthcare professionals should have productivity codes for community engagement activities that are equivalent to intramural activities. However, one CMHC has been able to develop a better coding system of medical services and procedures to reflect community engagement activities in close coordination with the supervising primary care center, most CMHCs lack such codes. For instance, an occupational therapist mentioned,

*“The community engagement activities should be equivalent to intramural activities when it comes to put them in the registry of provided services. For example, when you do an extramural activity, sometimes it does not have the same weight as an office-based service, it is not validated the same way”* (T3/CMHC1).

Clinicians also reported that having a clear organizational and reporting structure would improve the productivity approach and facilitate the feedback process. This proposed solution would likely address conflicts related to productivity goals for the supervising primary care center and the MoH, the former commonly emphasizes quantity of care, whereas the latter quality and community-based care.

### Promote Research to Assess Impact of the New Model of Care

Research emerged as a potential solution to assess the impact of activities that promote community involvement. Some participants were motivated to pursue research activities in the CMHCs, however the lack of training and time arose as barriers. While participants recognized the CMHM as a promising model of care, lack of systematized research on this matter results in limited knowledge about its impact on the healthcare system.

It was also reported that research of the CMHM would allow policy-makers to allocate resources more efficiently to community involvement activities that have a higher impact. Implementation of brief research workshops and time allowance were mentioned by clinicians as potential ways to promote research initiatives. Participants shared their interests to use quantitative approaches to assess impact and qualitative approaches to understand their target population. A participant nurse said:

*“There is lack of documentation, because we sometimes tell each other that we are pioneers on this, and we should do research, now there are other colleagues that conduct research on us. We should be more motivated, and start documenting our statistics, and epidemiology…”* (N2/CMHC1).

Overall, nurses, physicians and psychologists provided mostly data describing current community engagement activities and barriers to implementation whereas policy-makers elaborated primarily on barriers to implementation and potential solutions. This could be secondary to their clinical or leadership roles in the Peruvian mental health system. For instance, clinicians are expected to be more familiar with the day-to-day activities in the CMHCs, while policy-makers often deal with challenges and future steps of the implementation process.

## Discussion

Clinicians of the CMHCs and policy-makers included in the study reported that community engagement activities are common, however inconsistently being performed as part of a new strategy to provide mental healthcare in Peru. While there is substantial variation when reporting such activities in the literature, our study identified 5 community engagement activities. Similarly, a meta-review identified mental health awareness interventions, psychoeducation, skills training among lay community health workers, and rehabilitation and case management programs as primary community components of the CMHM in low- and middle-income countries (LMICs). Furthermore, community components are mainly delivered at homes and schools in addition to technological platforms.^36^

As shown in our study, substantial variation in the degree to which community components were integrated with primary care services have been also described in the literature.^36-39^ Overall, greater accessibility and acceptability compared to healthcare facilities, greater clinical effectiveness through ongoing contact and use of trusted local providers, family involvement and economic benefits have been described as main motivations to employ community components in LMICs.^36,40-42^

Our findings show that among community engagement activities, employing CMHWs and psychosocial clubs have been more consistently utilized across CMHCs. While employing community health workers (CHWs) has long been utilized in LMICs for people with medical conditions, there is still scarce literature regarding collaboration of CHWs with mental health clinics.^43-45^ Our study contributes with information about the role of CMHWs and challenges that impact the consistency and frequency of their activities in the community. Similarly, Mutamba et al in India, Pakistan and neighboring countries described that CMHWs aim to provide health education, screening, early detection and basic referral for people in the community.^46^

Our study showed that the curricula and training structure for CMHWs in Peru were inconsistent across the CMHCs. While the authors emphasize the importance to ensure consistent delivery of the model, there is still no agreement in the literature regarding their training structure. Training programs for CMHWs have ranged from a two-hour session to continuous supervision with booster sessions.^47^ The authors propose that topics such as mental health disorders, basic interventions for mental health promotion and prevention, and mental health first aid should be included in their training curriculum.

Furthermore, employing CMHWs is particularly important in mental health because it improves quality of care by contributing to patient-provider community, continuity of care, and consumer protection.^26,27^ While compensation of CHWs widely varies among countries, interventions should be actively implemented to retain these key staff members as part of the mental health workforce.^38,49^ Strategies to retain CMHWs have been discussed in the literature and are mainly focused on providing fair compensation, incentives and benefits to prevent their motivation to diminish over time.^50,51^ Conversely, our study evidenced that CMHWs in Peru do not receive any monetary compensation, and although the moral duty to do good for their communities appears to be their main driver to remain engaged and actively working, the lack of compensation is negatively impacting their motivation. The authors emphasize the importance of CMHWs, not only in LMICs, but also in high-income countries where certain populations maintain a strong sense of community; for instance, refugees or Hispanic populations.

Psychosocial clubs stood up as highly consistent community engagement activities in the CMHM in Peru. Although, there is a lack of a formal definition of a psychosocial club, our findings partially align with the Spanish Association of Neuropsychiatry, which defines psychosocial clubs as recreational activities to enhance motivation and interaction with the community.^52^ Psychosocial clubs in Peru have similarities with initiatives in other settings that aim to reintegrate people with mental illness into society in a meaningful way. For instance, Clubhouses have been implemented in the United States to allow people with mental illness to gain skills, locate a job, find housing, and pursue continuing education. Psychosocial clubs provide people with a safe environment to share their experiences, normalize their symptoms, and learn coping skills from their peers.^53-55^

Our study not only describes home visits, mental health campaigns, workshops and operational groups of peer-support as activities that are partially implemented across different CMHCs in Peru, but also identifies that they target different stages in the continuum of care (Figure 3). While promotion and prevention phases appear to be more commonly emphasized, introducing more treatment and recovery-oriented activities that promote community involvement may balance the model more evenly, and would prevent overloading certain phases of the continuum of care.

While the authors highlight the importance of the implementation of community engagement activities as part of a comprehensive model of care, we also recommend to consider the implementation of these activities independently, if logistical and financial challenges interfere with successful implementation of the entire new model of care. For instance, safety-net medical home initiatives, the National Alliance for Mental Illness psycho-education programs, and peer-support activities in Mental Health America (community-based nonprofit dedicated to addressing the needs of those living with mental illness) have shown that home visits, mental health campaigns, and peer-support activities individually, have positively impacted mental healthcare in the community.^56-59^

Our findings elucidated significant barriers to implementation related to limited financial resources, healthcare workers productivity evaluations and poorly defined catchment areas. As also evidenced in India and Colombia, we identified stigma as a strong barrier for the implementation of community involvement activities and access to mental healthcare.^60,61^ Stigma not only interferes with the ability to seek care by patients, but also results on hesitancy from the primary care provider to treat these patients, resulting in premature referrals to CMHC that can potentially overload the system.

Nonetheless, barriers found in our study could potentially be overcome by systematic interventions that participants have proposed. The proposed solutions were in the realm of education and training about the new model of care and mental health awareness. These barriers were also described in Latin American countries including Chile, Argentina, and Brazil, which have already started local training programs to train and nurture future generations of public health leaders. Although our study did not specifically described what a training program should include, examples of regional training initiatives entail having multidisciplinary teams of clinicians and researchers closely working with primary care and CMHC leaders to create a capacity-building platform for the next generation.^62,63^

Countries in South America, Europe and Africa have also implemented CMHMs.^10,64-66^ For instance, two decades ago, Chile began a mental health reform process to provide mental healthcare in the community. Changes in their model of care occurred slowly, and initially, challenges also arose including early discontinuation of treatment, inconsistent application of psychosocial interventions, and deficiencies regarding quantitative and qualitative development of human resources with relevant skills to community mental healthcare.^62,64^ Also, local initiatives to implement community mental health services in Argentina faced challenges such as inadequate productivity approach and evaluation, limited financial and human resources, which were also evidenced in our study in Peru.^63^

An important factor that contributed to the successful implementation of the CMHM in similar settings was the political commitment to narrow the gap for mental illness. The Chilean healthcare model has a particularly strong public health network, epidemiological research infrastructure, and advocacy by stakeholders, factors that are weaker in the Peruvian system. Therefore, the Peruvian MoH should continue to strengthen the primary care network, partnerships with academic institutions, and advocacy groups to continue the implementation process more effectively. Although efforts to engage key actors in the community have often fallen short in practice, there have been a few successful initiatives supporting the implementation of new facilities or activities. For instance, several CMHCs across the country have been successfully implemented due to collaborative partnerships among local municipalities, non-governmental organisations, churches, and healthcare institutions.^26,67-69^ Furthermore, key partnerships have resulted on local efforts to document certain aspects of mental health reform in the last several years.^70,71^ Notwithstanding, the progress related to the mental health gap in Peru is still limited.^62^

Despite regional initiatives to implement a CMHM, several Latin American countries still maintain a model of services based primarily on psychiatric hospitals and tertiary healthcare facilities, which results in the rapid consumption of their mental health budget.^62^ The development of cross-country regional initiatives related to the implementation of the CMHM have accelerated the pace of change resulting in mental health service development in neighbor countries. Chile, Peru and other countries in South America have also demonstrated that it is possible to positively and radically transform a psychiatric healthcare system and narrow the mental health gap.^63^

Our study elucidated details about community engagement activities, barriers and potential solutions to implementation specific for the Peruvian context that will help local policy-makers make recommendations to facilitate their implementation. While the authors highlight the importance of community engagement activities in the new model of mental healthcare, a rights-based approach calls also for transferring of planning and decision-making power to the individuals and communities that the health system is supposed to served. Service users and their families must be integrally involved in the policy-making and programming decisions.^72,73^ In fact, our study evidenced that the current role of patients and their families, and clinicians of CMHCs supporting community engagement activities have come a long way to successfully implement some of these activities despite ongoing barriers. Currently, the associations of relatives and mental health service users aim to play a key role in advocacy, stigma reduction and further expansion of mental health services.^74^

While we can situate our findings within several implementation theories to help us structure our observations, Normalization Process Theory should be further explored as a suitable approach to better identify factors that promote and inhibit routine incorporation of community engagement activities into daily practices.^75^

It is worth noting that opening dates of the CMHCs included in the study were approximately the same. Therefore, differences in the structure, consistency or content of community engagement activities were likely not secondary to their implementation date.

Finally, continuous and systematic implementation of community engagement activities will ultimately result in consistent and standardized services for people with mental illness, which would generate positive outcomes, improve efficiency and reduce costs.^76^ The implementation of an evaluation system will also provide policy-makers with valuable data to optimize the utilization of human and financial resources. Although, the authors believe that the CMHM is positively impacting the care of people with mental illness in Peru, there is a dearth of literature assessing the impact of the new model of care. The authors recommend the implementation of a national evaluation system as a next step in the process to assess impact of the CMHM.

There are a few notable limitations of this study. The three CMHCs included in the study may not represent those in other regions further away from Lima where they may have limited human resources and even less training in the new model of care. Thus, barriers and potential solutions to implementation of community engagement activities may differ from those that were included in this study. In addition, the survey focused only on those individuals who volunteered to participate. Future studies should include confidential reporting methods that would gain input from a wider array of staff members as well as from service users, their families and communities. The qualitative nature of this study does not allow the data and conclusions to be generalizable to other settings or countries, however it provides policy-makers with valuable information to improve the implementation process of the CMHC moving forward. The current study only assessed community engagement in the CMHM led by the Peruvian MoH, therefore our results do not reflect the current situation in other sectors of the healthcare system.

## Conclusion

Our findings suggest that activities to promote community engagement in the CMHCs are currently being conducted, however, clinicians and policy-makers perceive that their structure, frequency, and content are highly variable due to the lack of standardization of the new model of care. While several barriers to implementation were identified, rapid learning and implementation of an evaluation framework will be pivotal for the ultimate success of the CMHM. Finally, care for people with mental illness has changed, as such, a new standard of care has emerged to deliver mental healthcare in Peru.

## Acknowledgements

This paper presents independent research supported by Vanderbilt Institute for Clinical and Translational Research (VICTR): #VR51884. JAV was supported by the Overall Family Fellowship for International Research and Vanderbilt University School of Medicine MPH scholarships.

## Ethical issues

All study procedures were approved by the Comite de Etica (Ethics committee) of Universidad Peruana Cayetano Heredia in Lima, Peru and the Institutional Review Board (IRB) of Vanderbilt University Medical Center, Nashville, TN, USA. Written informed consent to take part in the interviews and for these to be audio recorded was obtained from all participants. Upon completion of the interviews, participants were provided with a snack. No monetary compensation was provided. A copy of the published manuscript translated into Spanish will be provided to the participants of each CMHC (clinicians) as well as to the policy-makers included in the study.

## Competing interests

Authors declare that they have no competing interests.

## Authors’ contributions

JAV wrote the first draft of the manuscript and coauthors contributed equally to subsequent drafts. All authors participated equally in the planning and development of the study protocol. JAV conducted field work, and collected and analyzed the data. FDC assisted during field work. All authors read and approved the final manuscript.

## Authors’ affiliations

^1^Department of Psychiatry and Behavioral Sciences, Vanderbilt University Medical Center, Nashville, TN, USA. ^2^Department of Pediatrics, Division of Infectious Diseases, and Vanderbilt Institute for Global Health, Vanderbilt University Medical Center, Nashville, TN, USA. ^3^Department of Health Policy, and Vanderbilt Institute for Global Health, Vanderbilt University Medical Center, Nashville, TN, USA. ^4^CRONICAS Center of Excellence in Chronic Diseases, Universidad Peruana Cayetano Heredia, Lima, Peru.

## Supplementary files


Supplementary file 1 contains the codebook developed and used in this study.

## Key messages

**Implications for policy makers**

- Promising advances in the care of patients with mental illness are likely to be accomplished in Peru after the implementation of the community mental health model (CMHM). However, an early evaluation of key aspects and barriers will provide policy-makers with evidence-based data to develop solutions for the ultimate success of the model.
- Identification and documentation of community engagement activities conducted in the community mental health centers (CMHCs) will allow policy-makers to allocate resources more efficiently to activities with higher impact in order to optimize the implementation process.
- Data showing barriers to implementation as well as potential solutions to improve community engagement activities will provide other low- and middle-income countries (LMICs) with important information about the implementation process, and valuable experiences and lessons learned to guide them through their own mental health reform process.

**Implications for public**

Community engagement activities stand out as a key component of the community mental health model (CMHM). As such, a better understanding of these activities will allow governmental and community-based institutions to increase investments intended to scale up these interventions to improve the care of people with mental illness in the community. Identifying the barriers to implementation and potential solutions will also allow advocacy groups, community-based organizations, and academic institutions to partner together to continue to improve mental healthcare delivery.
