# Supplementary material for: Perceptions of Community Involvement in the Peruvian Mental Health Reform Process Among Clinicians and Policy-Makers: A Qualitative Study
Source: Int J Health Policy Manag. 2019 Aug 21;8(12):711–22. doi: 10.15171/ijhpm.2019.68 (PMC6885866; doi:10.15171/ijhpm.2019.68)
Supplement: Supplementary file 1 — contains the codebook developed and used in this study. [file ijhpm-8-711-s001.pdf]

Supplementary file 1

1- Codebook

1 Community Engagement and mobilization activities	Code Frequency
1.1 current activities- community engagement	1
1.1.1 Home visits	59
1.1.2 GOAM	7
1.1.3 Mental health workshops	31
1.1.4 Mental health campaign	50
1.1.5 Psychosocial clubs	33
1.1.6 Training Community Mental Health Workers	35
1.2 Barriers	0
1.2.1 Infrastructure	45
1.2.2 Knowledge about the new model of care	77
1.2.3 Publicity	26
1.2.4 Transportation	31
1.2.5 Catchment Area	17
1.2.6 personnel	21
1.2.6.1 Stigma	21
1.2.6.1.1 Stigma - Community and patients	13
1.2.6.1.2 Stigma - Healthcare professionals	12
1.2.7 Productivity approach	43
1.2.8 Work Permit and organizational structure	42
1.3 Solutions/new ideas	0
1.3.1 Available transportation	19
1.3.2 Education in mental health	24
1.3.3 Training on CMHM for staff members in non-CMHC	34
1.3.4 Community health workers	3

1.3.5 Replication of model to other CMHC	25
1.3.6 University education on CMHM	9
1.3.7 Improve productivity evaluation	17
1.3.8 Partnership agreements with other organizations	19
1.3.9 Research to assess impact	12

1 Community Engagement and mobilization activities

Activities that entail processes of committed and responsible participation of the members and leaders of the community and its organizations. These aim to meet local community needs and play a role in the care of patients with mental health illness.

1.1 current activities- community engagement

Current activities performed in the CMHC that involved community engagement.

1.1.1 Home visits

Home visit performed by a health provider from the CMHC to provide care for patients or their families. Care includes promotion, prevention, treatment or recovery interventions.

1.1.2 GOAM

Operational group of peer support: Activities where patients discuss mental health topics, patients share experiences about how it is like to live with a mental health illness

1.1.3 Mental health workshops

talks and presentations led by staff members of the CMHC about mental health topics for patients and their families.

1.1.4 Mental health campaign

Type of media campaign which attempts to promote mental health awareness in the community. This may include involving schools, community-based organizations, etc. This also includes campaigns to make community aware of the existence of the center

1.1.5 Psychosocial clubs

Meetings with patients led by healthcare providers of the CMHC, they organize activities in the community in order to observe how patients do in an recreational or social environment. This may include going to movie theater, or grocery stores, etc.

1.1.6 Training Community Mental Health Workers

Activities that involve training community health workers on promotion, prevention and identification of mental health conditions. A community health worker is a trusted member that has a close understanding of the community served.

1.2 Barriers

Problems that interfere with expected performance for community engagement activities

1.2.1 Infrastructure

Lack of areas and spaces in the CMHC to hold community engagement activities. Materials that are needed in the center to appropriately perform activities.

1.2.2 Knowledge about the new model of care

Lack of knowledge and training in the community Mental Health Center among employees. This also includes knowledge about referral process.

1.2.3 Publicity

Lack of public visibility or awareness for the services and existence of a community mental health center.

1.2.4 Transportation

Problems with transportation availability to visit patients

1.2.5 Catchment Area

Unclear area of the city and population that the community mental health center should serve

1.2.6 personnel

Limited personnel to do activities in the center and outside.

1.2.6.1 Stigma

Negative attitudes and/or beliefs toward people who have a mental health condition or mental health care in general.

1.2.6.1.1 Stigma - Community and patients

Stigma on patients and/or community

1.2.6.1.2 Stigma - Healthcare professionals

Stigma on healthcare professionals

1.2.7 Productivity approach

Inadequate assessment of productivity which does not reflect importance of extramural activities and expectations

1.2.8 Work Permit and organizational structure

Lack of work permit in the center. Lack of having a clear organizational structure, including knowing who is the director of the center and his functions. This also includes work instability.

1.3 Solutions/new ideas

Potential solutions and proposed ideas to overcome barriers that currently exist.

1.3.1 Available transportation

Have transportation available for community engagement activities. This could include provided by other organization or owned. This also include having a small budget for daily expenses in the centers.

1.3.2 Education in mental health

Continue to provide education in mental health to general public to decrease stigma. This includes knowledge about their rights as patients.

1.3.3 Training on CMHM for staff members in non-CMHC

Continuous training and employment of CMHM for primary care clinics, and other healthcare institutions.

1.3.4 Community health workers

Emphasize the work with community health workers in order to get closer to the community

1.3.5 Replication of model to other CMHC

Replicate activities that are being conducted appropriate to other CMHCs in order to get experience through modeling. This includes training sessions for healthcare workers in the CMHCs. Peer learning.

1.3.6 University education on CMHM

Include training on the CMHM into curriculum for healthcare providers.

1.3.7 Improve productivity evaluation

change current productivity assessment to emphasize extramural activities. This also includes clarifying the organizational structure and bureaucratic steps

1.3.8 Partnership agreements with other organizations

To develop partnership agreements with community-based organizations or healthcare institutions and CMHC

1.3.9 Research to assess impact

Emphasize and implement the development of research projects in the CMHCs to assess impact, and to evaluate the current model.
